# Spatial Co-Occurrence and Activity Patterns of Mesocarnivores in the Temperate Forests of Southwest China

**DOI:** 10.1371/journal.pone.0164271

**Published:** 2016-10-10

**Authors:** Hongliang Bu, Fang Wang, William J. McShea, Zhi Lu, Dajun Wang, Sheng Li

**Affiliations:** 1 School of Life Sciences, Peking University, Beijing, 100871, China; 2 Center for Systems Integration and Sustainability, Michigan State University, East Lansing, Michigan, 48823, United States of America; 3 Smithsonian Conservation Biology Institute, Front Royal, Virginia, 22630, United States of America; University of Southern Queensland, AUSTRALIA

## Abstract

Understanding the interactions between species and their coexistence mechanisms will help explain biodiversity maintenance and enable managers to make sound conservation decisions. Mesocarnivores are abundant and diverse mid-sized carnivores and can have profound impacts on the function, structure and dynamics of ecosystem after the extirpation of apex predators in many ecosystems. The moist temperate forests of Southwest China harbor a diverse community of mesocarnivores in the absence of apex predators. Sympatric species tend to partition limited resources along time, diet and space to facilitate coexistence. We determined the spatial and temporal patterns for five species of mesocarnivores. We used detection histories from a large camera-trap dataset collected from 2004–2015 with an extensive effort of 23,313 camera-days from 495 camera locations. The five mesocarnivore species included masked palm civet *Paguma larvata*, leopard cat *Prionailurus bengalensis*, hog badger *Arctonyx collaris*, yellow-throated marten *Martes flavigula*, and Siberian weasel *Mustela sibirica*. Only the masked palm civet and hog badger tended to avoid each other; while for other pairs of species, they occurred independently of each other, or no clear pattern observed. With regard to seasonal activity, yellow-throated marten was most active in winter, opposite the pattern observed for masked palm civet, leopard cat and hog badger. For diel activity, masked palm civet, leopard cat and hog badger were primarily nocturnal and crepuscular; yellow-throated marten was diurnal, and Siberian weasel had no clear pattern for most of the year (March to November), but was nocturnal in the winter (December to February). The seasonal shift of the Siberian weasel may be due to the high diet overlap among species in winter. Our results provided new facts and insights into this unique community of mesocarnivores of southwest China, and will facilitate future studies on the mechanism determining coexistence of animal species within complex system.

## Introduction

How ecologically similar species coexist has been a key question in ecology, and is crucial to understanding community diversity [1, 2]. Intraguild competition plays an important role in shaping species’ ecological niches by affecting their ability to access limited resources [3, 4]. To mitigate the negative impact of interspecific competition, species often partition resources along three main niche dimensions (time, food and space), which results in niche differentiation [5, 6]. Information on species’ niche differentiation will help scientists understand the capacity of species to coexist and also benefit conservation and management of biodiversity, especially within communities that have lost apex predators [7, 8].

One of the common outcomes following the loss of apex predators is the dramatic increase in the abundance of mesocarnivores, which then depress numbers of smaller prey species, a phenomenon known as “mesopredator release” [9, 10]. Mesocarnivores in terrestrial systems are mid-trophic level predators within a specific range of body weights (e.g., 1 to 15 kg) [11, 12]. Compared with apex predators, mesocarnivore guilds generally have higher species’ richness, overall abundance, and exhibit a more diverse resource and habitat use [13]. Evidence of mesopredator release following apex predator removal has been demonstrated in both terrestrial [14, 15] and aquatic [16, 17] ecosystems. For example, Crooks and Soule [9] found that the decline and disappearance of coyote *Canis latrans*, in conjunction with the effects of habitat fragmentation, increased the distribution and abundance of smaller predators (i.e., domestic cat *Felis catus*, Virginia opossum *Didelphis virginiana* and northern raccoon *Procyon lotor*), which increased predation rates on their avian prey. Understanding the mechanisms that maintain diverse mesocarnivore communities would enable decision makers to understand the consequences of their management activities to control individual species.

The Minshan Mountains of Southwest China harbor a unique temperate forest ecosystem with the highest biodiversity in the Northern Hemisphere [18, 19]. The native carnivore species include Asiatic black bear *Ursus thibetanus*, giant panda *Ailuropoda melanoleuca*, Asiatic golden cat *Catopuma temminckii*, leopard cat *Prionailurus bengalensis*, red fox *Vulpes vulpes*, hog badger *Arctonyx collaris*, Chinese ferret badger *Melogale moschata*, yellow-throated marten *Martes flavigula*, Siberian weasel *Mustela sibirica*, least weasel *Mustela nivalis* and masked palm civet *Paguma larvata* ([20]; Li S. unpublished data). Except for the Asiatic black bear and giant panda, the other species are considered mesocarnivores. Although the carnivore guild remains diverse, apex predators (e.g., tiger *Panthera tigris*, dhole *Cuon alpinus* and leopard *Panthera pardus*) have been extirpated from many sites during the recent decades [21–24]. An increase in mesocarnivore populations within the region could increase predation on local terrestrial birds and small mammals. Endangered Phasianid species have been reported as prey for leopard cats [25, 26], masked palm civets [27], and yellow-throated martens [28] and Asiatic golden cat (Yao M., unpublished data). Masked palm civets and Siberian weasels are also identified as nest predators for ground-dwelling birds in southwest China [29]. Therefore, it is necessary to determine the current status of this mesocarnivore community as the first step toward assessing their impacts on sympatric species.

Previous studies on the diet of these mesocarnivores in Southeast Asia and China (Table 1) showed that leopard cats and Siberian weasels consumed primarily small mammals [31, 33–35]; both masked palm civet and yellow-throated marten fed on fruits and small mammals, and switched prey according to the seasonal availability of fruits [27, 28]; and hog badgers fed predominantly on earthworms and fruits [32]. To facilitate coexistence, we hypothesized that species with high diet overlap (e.g., leopard cats and Siberian weasels; masked palm civets and yellow-throated martens) would exhibit low overlap in their activity patterns. In winter when fruits are absent and all species rely on small mammals [26–28, 31–33], we predict lower activity overlap during this period due to the increase of diet overlap. Studies with domesticated masked palm civets found that they lowered their metabolism and activity levels from December to February [36]. Hog badgers are also reported to hibernate in the winter [32]. Such habits reduce the number of species competing for food resources in winter. However, Zhou et al. [37] concluded that wild masked palm civets did not hibernate during winter.

**Table 1:** Body size and diet of the five common mesocarnivores according to studies from China and Southeast Asia. Data of body sizes are from Smith et al. [30]. Relative importance of food sources was classified into three categories: P, primary; S, secondary; and O, occasional.

Species	Body size (kg)	Invertebrates	Smaller vertebrates		Plants	Source
			Others (reptiles, amphibians, birds)	Small mammal		
Masked palm civet^a^	3–7	O	S	P	P	Zhou et al. [27]
Yellow-throated marten^a^	0.8–2.8		O	P		Chiang et al. [31]
		O	S	P	S	Zhou et al. [28]
Hog badger	9.7–12.5	P	O	O	S	Zhou et al. [32]
Leopard cat	1.5–5		S	P		Shehzad et al. [25]
			S	P	P^b^	Xiong et al. [26]
		S	O	P		Grassman et al. [33]
		O	S	P		Rajaratnam et al. [34]
Siberian weasel	0.5–1.2	S	O	P		Chiang et al. [31]

a According to Zhou et al. [27, 28], masked palm civets and yellow-throated marten feed primarily on fruits and small mammals, and they switch their diet in response to the seasonally available fruits.

Xiong et al. [26] observed high occurrence of plant material in scats of leopard cats using DNA-based method.

Another factor complicating any examination on species coexistence from activity periods is the degree of spatial overlap between sympatric species. For instance, microhabitat differences in the distribution of species would allow co-existence of species that seemingly overlap in diet and activity. Therefore, sampling would need include a measure of co-occurrence and account for the potential of significant differences in detectability between the species [38].

Here we present the first study on the co-occurrence and activity patterns of this mesocarnivore guild using empirical camera-trap data. The objectives of this study were to: 1) measure the co-occurrence patterns between species; 2) determine the diel and monthly activity patterns of each species; 3) quantify the temporal overlap between species; and 4) explore if activity patterns shift during the resource-limited winter period.

## Material and Methods

### Study Area

**Fig 1:**
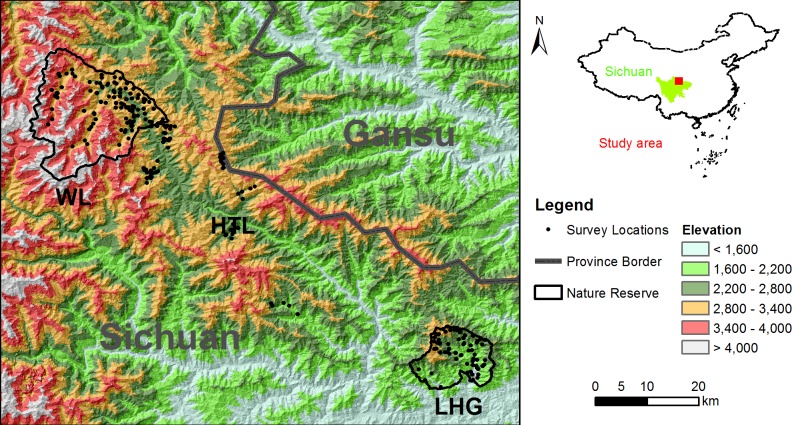
Study area and survey locations distributed in Minshan Mountains, Sichuan, China. The area includes 2 nature reserves (WL and LHG), and the unprotected area connecting established reserves (HTL, a proposed landscape corridor for giant panda).

The Minshan Mountains (31°24′ – 34°34′N and 102°38′ – 105°64′, Fig 1) are located in Sichuan and Gansu Provinces, along the north edge of the Southwest China biodiversity hotspot [18]. This area is characterized by rugged terrain with high mountains and deep valleys. Along the elevation gradient, the major vegetation types include alpine meadow (> 3,200 m), conifer forest (2,800–3,200 m), conifer-deciduous mixed forest (2,400–2,800 m), broadleaf forest (< 2,400 m) and early successional fields or agriculture that are distributed along river valleys [20]. This study was conducted across a broad elevation (1,300–3,500 m) within northern Minshan Mountains, involving two nature reserves, i.e., Wanglang National Nature Reserve (WL) and Laohegou Nature Reserve (LHG), and Huantulaing (HTL), an unprotected area connecting the reserves (Fig 1). Field survey was approved by Sichuan Forest Department (SFD). No IACUC permission was required by our institutions because there was no direct contact between the survey staff (remote camera with infrared flash) and the animals.

### Data Collecting

We surveyed mammals in our study area as part of a long-term monitoring program in the year of 2004–2006, 2008 and 2011–2015. Within each study area we generated a grid of 1 km × 1 km cells, as potential sampling blocks. To optimize animal detections and convenience, we placed one to two cameras in suitable habitat with animal sign or along game trails and little human disturbance within each cell. After about four to six weeks, remote cameras were relocated to another blocks [39]. Cameras were mounted on trees at 40–60 cm above ground level, set to work 24 h per day, and all survey stations were baited with commercial carnivore scent lure (Carman’s Magna-Glan Lure, Russ Carman, New Milford, Pennsylvania) to slow animal movement and ensure sufficient reaction time for the infrared sensor [40]. During any single survey period we positioned the cameras >300 m of each other to reduce potential spatial autocorrelation among neighboring locations (for details see Li et al. [20]).

### Data Analysis

#### Spatial co-occurrence patterns

We investigated species co-occurrence pattern following the modeling protocol presented by MacKenzie et al. [38] in program PRESENCE [41]. We constructed detection histories by dividing the camera-trapping duration at each survey station into 15-day segments. For each segment, a species was considered “detected” if the species were photographed, “not detected” otherwise. We fitted 16 models for each species pair (S1 Table) and draw inference about species’ co-occurrence pattern according to the species interaction factor (SIF, $\hat{\gamma }$) estimated from the top models (ΔAIC < 2) [42]. Values of $\hat{\gamma }$ < 1 would suggest species avoidance, while $\hat{\gamma }$ > 1 would suggest species co-occur more frequently than expected, and $\hat{\gamma }$ = 1 would suggest species occur independently [38].

We included 2 variables that might affect detection probabilities of species: camera trap model and scent lure persistence. We classified the camera trap models according to the trigger delay into fast (≤ 1 s), or slow (> 1 s) [40]. We divided the time since scent lure application during each survey into 0–15, 16–30, 31–45, >45 days.

For this analysis we only included data from the survey in 2011 to 2015, because they have covered most of the camera stations surveyed before. We also excluded the winter data in the modeling because of the hibernation-like behavior of masked palm civets during winter described below, which obviously violated the assumption of closed occupancy status [43].

#### Seasonal activity

In the analysis of activity, we pooled data together collected from all the years to achieve sufficient sample size (S1 File) for a robust comparison and statistical examination. We assumed no abrupt changes of animals’ activity patterns across our survey years because 1) our surveys happened many years after the loss of apex predators [21–24] which might affect animals’ activity; and 2) we placed our cameras at locations with little human disturbance to evade the effect of varying human activities.

We quantified the relative activity indices (RAI) in each month as the number of detections per 1,000 camera-days; therefore, the RAI in month *j* was the ratio of number of detections and camera-days in month *j* multiplied by 1,000 camera-days. We defined a detection as consecutive pictures of the same species > 0.5 h apart [44], and multiple individuals in one photograph were considered as a single detection of the species [45].

#### Diel activity patterns

An animals’ activity patterns are constrained by endogenous rhythms which allow it to cope with the external environment, such as seasonal variation in sunrise and sunset [46]. In our study area, the maximum variation in sunrise (i.e., between the earliest and latest sunrise) is approximately 2 hours. To account for the seasonal variation of sunrise and sunset, we standardized our observations by transforming the clock-recorded time of each detection to a relative sun time corresponding to the actual sunrise and sunset (sensu Nouvellet et al. [47]) and the relative sunrise and sunset were set at 06:00 and 18:00, respectively.

We considered detections of a species as a random sample derived from the distribution of its underlying continuous activity, and activity graphs describe the probability of a species being detected at any particular interval of the day [48]. Based on the camera detections, the daily activity pattern of each species was estimated using kernel density estimation according to the approach proposed by Ridout and Linkie [49] with “*overlap*” package [50] in R software [51] with a smoothing parameter of 1.0.

We examined species’ selectivity to time periods by comparing use to availability of each time period [52]. We classified the diel cycle into crepuscular, diurnal, and nocturnal periods. We defined the crepuscular hours as periods from the start of morning astronomical twilight to sunrise, and from sunset to the end of astronomical twilight [53]. We defined diurnal as the hours from sunrise to sunset, and nocturnal as the remaining time. To see if species’ activity was predominately classified as crepuscular, diurnal, or nocturnal, we calculated selection ratios of use to availability to each time period by each species following Manly et al. [52]:

$$w_{i}=o_{i}/{\hat{\pi }}_{i}$$

*w*_*i*_ is the selection ratio for the period *i*; o_*i*_ is proportion of detections in period *i*; ${\hat{\pi }}_{i}$ is proportions of length in period *i* to the length of all periods. *w*_*i*_ > 1 indicates that the time period is selectively used more than availability; *w*_*i*_ < 1 indicates the time period is avoided [54].

We used χ^2^ tests to determine if species used the three time periods non-randomly. If the species used the time periods non-randomly, we used a randomization procedure to test how the pattern deviated from random. We regarded detections in crepuscular, diurnal and nocturnal periods as a multinomial distribution and the probability in each class was determined by the length of that period. We calculated the length of periods as sum of all camera trapping days at all sites. We compared the observed detection number with the distribution obtained by repeating the randomization procedure 10,000 times.

#### Diel temporal overlap

We measured the overlap for all species pairs, using the *coefficient of overlap*, $\hat{\Delta }$, which ranges from 0 (no overlap) to 1 (complete overlap) [49]. The coefficient is defined as the area under the curve which is formed by taking the minimum of the two density functions at each time point [48]. The confidence interval of the overlap was obtained by bootstrapping 10,000 samples from the estimated probability density functions of each species. We also used temporal overlap of a species’ diel activity pattern in different seasons to test if activity shifted between seasons [45]. We expected a low $\hat{\Delta }$ value if species altered their activity pattern between seasons. Calculation of temporal overlaps and their confidence intervals were conducted with “*overlap*” package [50] in R software [51] with a smoothing parameter of 1.0.

## Results

From 2004 to 2015, we accumulated 23,313 camera-days from 495 survey locations (Table 2; S2 Table, S2 File). We obtained at least 100 detections for masked palm civet, leopard cat, hog badger, yellow-throated marten and Siberian weasel (Table 2; S1 File). Other detected mesocarnivores included Asiatic golden cat, Chinese ferret badger and red fox, but detections for these three species were too few for further analysis (S2 Table).

**Table 2:** Sampling efforts and numbers of independent detections of carnivore species during the camera-trap survey in Minshan Mountains, Sichuan, China from 2004 to 2015.

	Warm season	Winter	Total
Camera-days	20,490	2,823	23,313
Species detections			
Masked palm civet	136	0	136
Leopard cat	115	7	122
Yellow-throated marten	75	26	101
Siberian weasel	208	43	251
Hog badger	203	5	208

### Species co-occurrence patterns

For masked palm civet and hog badger, value of species interaction factor (SIF, $\hat{\gamma }$±SE = 0.60±0.19) estimated from the only existed top model (ΔAIC < 2) indicated avoidance. For 5 species pairs (i.e. leopard cat and masked palm civet, yellow-throated marten with the other 4 species), there were no clear patterns according to the models. There were ≥2 top models and no specific hypotheses ($\hat{\gamma }$ = 1 and $\hat{\gamma }$ ≠ 1) received overwhelmingly support (Table 3). For the other species pairs, the top models suggested that species occurred independently of each other (Table 3).

**Table 3:** Top models and estimation of species interaction factor (SIF, $\hat{\gamma }$) for each species pair from two species occupancy modeling. The species abbreviations of species’ names are LC-leopard cat, MPC-masked palm civet, HB-hog badger, SW-Siberian weasel, and YTM-yellow-throated marten. *K* is the number of estimated parameters in the model and ΔAIC is the absolute difference in AIC values relative to the model with the smallest AIC. The term “S” in parentheses denotes that the occupancy probability or detection probability of species were estimated separately for each species, and “·” indicates that the parameter is constant. Absence of γ(·) in the model notation implies that γ = 1 and absence of *r*(S) implies *r*(S) = *p*(S). “Lr” refers to scent lure persistence; and “Cam” refer to camera trap models.

Species pairs	$\hat{\gamma }$±SE	ΔAIC < 2 models	ΔAIC	K	-2*LL
MPC, HB	0.60±0.19	ψ(S)γ(.)*p*(S+L*r*+Cam)*r*(S+Lr+Cam)	0.00	18	991.76
MPC, LC	1.00±0.00	ψ(S)*p*(S)*r*(S)	0.00	7	930.15
	0.88±1.26	ψ(S)γ(.)*p*(S)*r*(S)	1.99	8	930.14
MPC, YTM	1.00±0.00	ψ(S)*p*(S)*r*(S)	0.00	7	825.42
	1.00±0.00	ψ(S)*p*(S+Lr)*r*(S+Lr)	0.40	12	815.82
	0.70±0.29	ψ(S)γ(.)*p*(S)*r*(S)	1.14	8	824.56
	0.63±0.28	ψ(S)γ(.)*p*(S+Lr)*r*(S+Lr)	1.14	13	814.56
LC, YTM	1.00±0.00	ψ(S)*p*(S)*r*(S)	0.00	7	807.07
	0.79±0.49	ψ(S)γ(.)*p*(S)*r*(S)	1.84	8	806.91
YTM, HB	1.00±0.00	ψ(S)*p*(S)*r*(S)	0.00	7	901.37
	0.77±0.40	ψ(S)γ(.)*p*(S)*r*(S)	1.69	8	901.06
YTM, SW	1.52±0.32	ψ(S)γ(.)*p*(S+Lr+Cam)	0.00	12	933.08
	1.00±0.00	ψ(S)*p*(S+Lr+Cam)	0.91	11	935.99
SW, HB	1.00±0.00	ψ(S)*p*(S+Lr+Cam)*r*(S+Lr+Cam)	0.00	17	1085.96
MPC, SW	1.00±0.00	ψ(S)*p*(S+Cam+Lr)*r*(S+Cam+Lr)	0.00	17	1057.45
LC, HB	1.00±0.00	ψ(S)*p*(S+Cam+Lr)*r*(S+Cam+Lr)	0.00	17	972.85
LC, SW	1.00±0.00	ψ(S)*p*(S+Lr)*r*(S+Lr)	0.00	12	1055.44
	1.00±0.00	ψ(S)*p*(S+Lr)	1.50	8	1064.94

### Species’ seasonal activities

**Fig 2:**
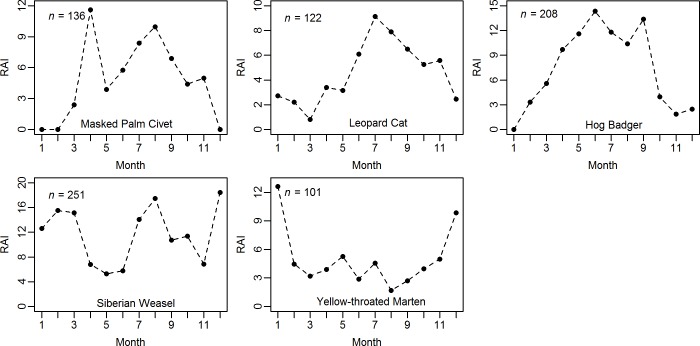
Relative activity indices (RAI, number of detections per 1,000 camera-days) in each month and number of total detections (*n*) for each mesocarnivores in Minshan Mountains, Southwest China.

The survey efforts in each month were 1,944±763 (mean±SD) camera-days. The minimum effort was 813 camera-days in December; and maximum effort was 2,848 camera-days in May. For all the species, the detections in each month were distributed disproportionately to the camera-days (S3 Table). The masked palm civet, leopard cat and hog badger showed higher activity (high RAI) from June to September and lower activity (low RAI) from November to March (Fig 2). We did not detect the masked palm civet from December to February, even with a large survey effort (Fig 2; Table 2). In contrast, the yellow-throated marten was more active in December and January than other months. Siberian weasels displayed two annual high-activity periods (July to September, and December to March).

### Diel Activity patterns

Since masked palm civets were not detected from December to February, we estimated the diel activity patterns for the winter (December-February) period separately from the rest of the year (henceforth called warm season).

**Fig 3:**
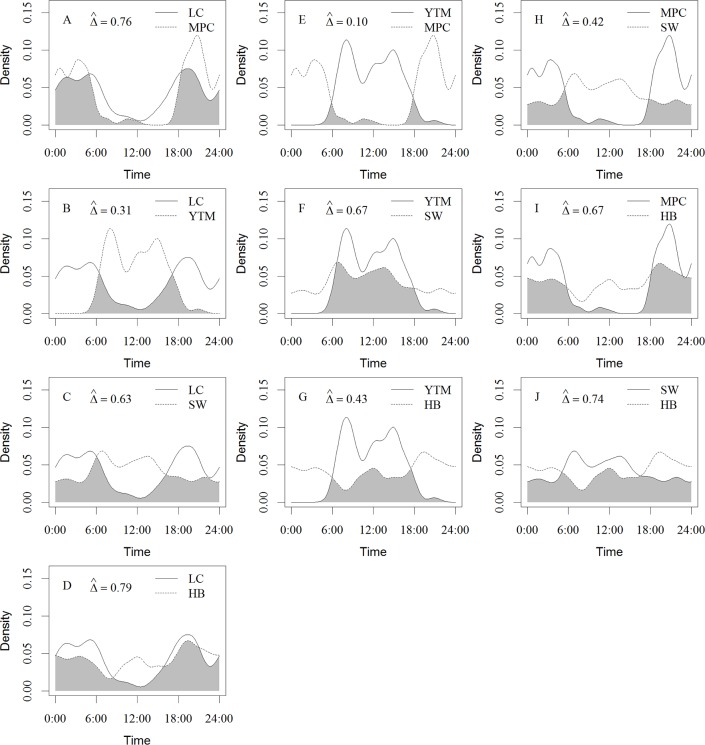
Diel activity patterns and temporal overlap in the species of mesocarnivore guild during warm season (March to November) in Southwest China. The y-axis is the “Kernel Density Estimates”. The overlap area was denoted in grey. The species abbreviations of species’ names are LC-leopard cat, MPC-masked palm civet, HB-hog badger, SW-Siberian weasel, and YTM-yellow-throated marten.

In the warm season, all species except the Siberian weasel exhibited nonrandom use of crepuscular, nocturnal, and diurnal periods (Table 4; Fig 3). The masked palm civet, was detected more than expected during the nocturnal (*P* = 0.000) and crepuscular (*P* < 0.001) periods, and less than expected during the diurnal period (*P* = 0.000). The leopard cat was also detected more than expected during the nocturnal (*P* < 0.001) and crepuscular (*P* < 0.001) periods, and less than expected during the diurnal period (*P* = 0.000). For the hog badger, we detected it more than expected during the nocturnal (*P* < 0.001) and crepuscular (*P* = 0.047) periods, and less than expected during the diurnal hours (*P* < 0.001). Yellow-throated martens were detected mostly during the diurnal hours (*P* = 0.000), and infrequently detected during the nocturnal (*P* = 0.000) and crepuscular (*P* = 0.012) periods.

**Table 4:** Number of detections *n* (selection ratio *w*) and the random use test of the crepuscular, nocturnal and diurnal periods given their availability by the mesocarnivores in warm (Mar.-Nov.) and winter (Dec.-Feb.) seasons in Minshan Mountains of Sichuan, China.

Species	*n* (*w*) in time periods			Random use test (χ^2^, *df* = 2)
	Crepuscular	Diurnal	Nocturnal	
Warm season (Mar.-Nov.)				
Masked palm civet	32 (1.88)	5 (0.07)	99 (2.17)	139.57, *P* < 0.001
Leopard cat	29 (2.01)	28 (0.45)	58 (1.51)	43.32, *P* < 0.001
Yellow-throated marten	3 (0.32)	71 (1.75)	1 (0.04)	50.53, *P* < 0.001
Siberian weasel	25 (0.96)	127 (1.13)	56 (0.80)	4.66, *P* = 0.097
Hog badger	34 (1.34)	77 (0.70)	92 (1.35)	21.03, *P* < 0.001
Winter season (Dec.-Feb.)				
Yellow-throated marten	1 (0.32)	23 (2.03)	2 (0.17)	21.47, *P* < 0.001
Siberian weasel	5 (0.98)	11 (0.59)	27 (1.41)	6.36, *P* = 0.042

In the winter, we could only compare the Siberian weasel and yellow-throated marten due to small sample size of the other species (Table 2). Yellow-throated martens were detected mostly during diurnal hours (*P* = 0.000), and infrequently during nocturnal period (*P* = 0.000). Siberian weasel was detected more than expected during the nocturnal period (*P* = 0.011), and less than expected during the diurnal period (*P* = 0.012).

### Diel temporal overlap

**Fig 4:**
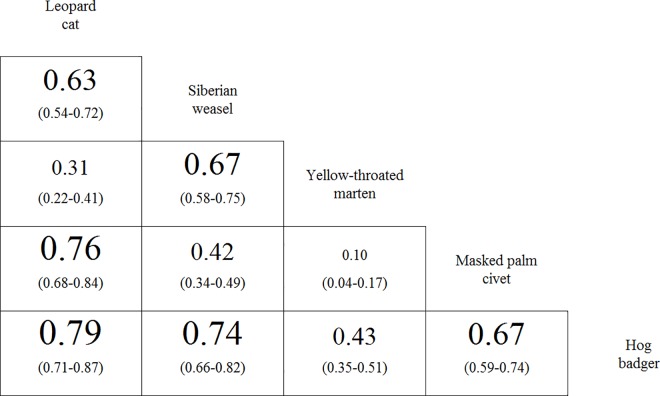
Diel activity overlap and confidence intervals for each species pair among the mesocarnivores during warm season in Minshan Mountains, Southwest China. The species are sorted from highly carnivorous (leopard cat) to omnivorous (Hog badger). The font size of the overlap value is proportional to its value.

In the warm season, the leopard cat and hog badger had the highest temporal overlap ($\hat{\Delta }$ = 0.79; Figs 3D and 4), followed by the species’ pairs of the leopard cat and masked palm civet ($\hat{\Delta }$ = 0.76; Fig 3A), and the Siberian weasel and hog badger ($\hat{\Delta }$ = 0.74; Fig 3J). The lowest temporal overlap was between the masked palm civet and yellow-throated marten ($\hat{\Delta }$ = 0.10; Fig 3E), followed by the leopard cat and yellow-throated marten ($\hat{\Delta }$ = 0.31; Fig 3B).

**Fig 5:**
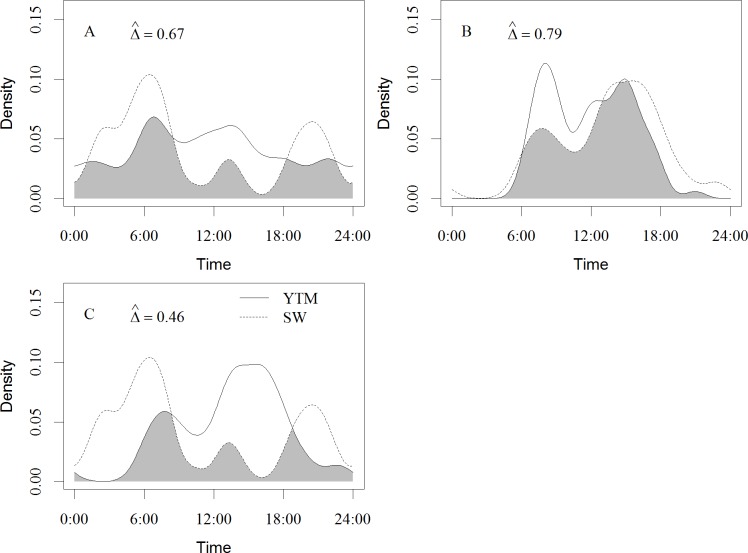
Diel activity patterns and overlap of yellow-throated marten (YTM) and Siberian weasel (SW) in the winter (December to February) and between seasons in Southwest China. The overlap area was denoted in grey. (A) overlap for Siberian weasel between seasons; (B) overlap for yellow-throated marten between seasons; (C) overlap between Siberian weasel and yellow-throated marten in winter.

The diel activity pattern of Siberian weasel was differed from warm season to winter ($\hat{\Delta }$ = 0.67 (95% CI: 0.55–0.79); Figs 4 and 5A). Siberian weasels were more active during the nocturnal period in winter than in the warm season. The daily temporal overlap of yellow-throated marten and Siberian weasel during the warm season ($\hat{\Delta }$ = 0.67; Fig 3F) decreased by 31% in the winter ($\hat{\Delta }$ = 0.46 (95% CI: 0.27–0.63); Fig 5C).

## Discussion

Among the five mesocarnivores examined, we only observed species avoidance between masked palm civet and hog badger from the two-species occupancy modeling; the rest pairs of species occurred independently, or no clear pattern observed, indicating no significant inferences (competition or predation) within most of the species. One way to reduce competition is through non-overlapping activity patterns. The masked palm civet, leopard cat and hog badger were more active in the nocturnal and crepuscular periods, the yellow-throated marten was diurnal, and the Siberian weasel lacked a distinct daily activity pattern in the warm season, but became nocturnal in the winter.

This study is the first estimate of activity pattern for the hog badger. The results for masked palm civet are consistent with previous reports [55, 56] except Zhou et al. [37], who also observed activity peak between 08:00 and 12:00 by analyzing data from radio-collared individuals. Yellow-throated martens showed a diurnal activity pattern in our study, which is similar with Chiang et al. [31], but in contrast with Grassman et al. [57] who found some nocturnal activities. The nocturnal/crepuscular activity pattern of leopard cat we recorded was consistent with some reports [56, 58], but in contrast to arrhythmic activity reported by several researchers in Thailand [33, 35, 59, 60]. Rabinowitz [59] and Grassman [35] contended that an arrhythmic activity pattern resulted from leopard cats optimizing use of a diverse community of nocturnal and diurnal prey, whereas Rajaratnam [58] believed that nocturnal activity benefited leopard cats preying upon nocturnal rodents. The variation of activity patterns across different sites may be attributed to the broad flexibility and adaptability of leopard cats.

For yellow-throated marten and masked palm civet, which probably have high diet overlap by consuming primarily on seasonally available fruits and small mammals ([27, 28]; but see Chiang et al. [31]); we observed low diel activity overlap and an opposite pattern of seasonal activity (Fig 2).

Some species exhibited variable seasonal activity, with both the hog badger and masked palm civet being rarely detected in the winter. We detected no civets and only five times of hog badger during December to February, and that conformed with previous studies which found masked palm civets had hibernation-like behaviors from December to February [36]. We speculated that such habits might be a result of expending less energy in the resource-limited winter, which eliminated a competitor to the martens [32, 36].

For species pairs who had high activity overlap, we also observed more difference on diet (see the lower-left corner in Fig 4). Masked palm civet and hog badger tended to avoid spatially (Table 3), which probably resulted from their relatively high activity overlap and similar food resources (Fig 4). There were also species pairs who overlapped on both diet and activity pattern, but no spatial segregation was observed, for example Siberian weasel with leopard cat and yellow-throated marten (Table 3; Fig 4). Such result implied there might be other niche dimensions not considered in our study. For instance, benefiting from their smaller body size, Siberian weasel might prey on rodents in the burrows and runways [61]. Such advantage might promote coexistence between these two species.

We observed an activity shift by Siberian weasel from random to nocturnal activity pattern between warm season and winter (Fig 5). One consequence of this shift was reduced temporal overlap between Siberian weasel and yellow-throated marten in winter (Figs 4 and 5). Specifically, in winter when no fruits were available, yellow-throated marten switched to prey on small mammals which were also the main food resource of Siberian weasel [31]. Similarly, Chiang et al. [31] observed that Siberian weasels exhibited nocturnal activity in southern Taiwan, especially when sympatric with yellow-throated martens. These researchers also speculated that the Siberian weasels adjusted their activity patterns to reduce their temporal overlap with the more dominant martens. Our results are consistent with the hypothesis that competition exists in the resource-limited winter period. We speculate that Siberian weasels shift to nocturnal activity to reduce encounters with the larger body-sized yellow-throated marten.

Considering the population decline and distribution contraction of apex predators across the world [7], and the potential impacts of irrupted mesocarnivores on ecosystems [9, 10, 12, 13]; we advocate more studies on mesocarnivores’ interactions and their impacts on sympatric species. In conclusion, our results reveal diverse co-occurrence patterns, diel and seasonal activities among a rich community of mesocarnivores, which may promote their coexistence and inform future studies of predator interactions.

## Supporting Information

S1 File Camera-trapping detections of mesocarnivores in Minshan Mountains, Southwest China. (XLSX)

S2 File Survey effort of each camera-trapping station in Minshan Mountains, Southwest China. (XLSX)

S1 Table Fitted models for each species pair in the two species occupancy modeling. The term “S” in parentheses denotes that the occupancy probability or detection probability of species were estimated separately for each species, and “·” indicates that the parameter is constant. Absence of γ(·) in the model notation implies that γ = 1 and absence of *r*(*S*) implies *r*(*S*) = *p*(*S*). “Lr” refers to the covariate scent lure persistence; and “Cam” refer to camera trap models.
(DOCX)

S2 Table Number of survey locations, survey efforts, and detections (number of locations which detected the species) for each species in the remote camera survey from 2004 to 2015 in Minshan Mountains, Southwest China. (DOCX)

S3 Table Number of detections, camera days in each month for all 5 species and *χ*^2^-test results on detection distribution to camera-days in the remote camera survey from 2004 to 2015 in Minshan Mountains, Southwest China. (DOCX)
