# Supplementary material for: Spatial Co-Occurrence and Activity Patterns of Mesocarnivores in the Temperate Forests of Southwest China
Source: PLoS One. 2016 Oct 10;11(10):e0164271. doi: 10.1371/journal.pone.0164271 (PMC5056745; doi:10.1371/journal.pone.0164271)
Supplement: S1 Table — The term “S” in parentheses denotes that the occupancy probability or detection probability of species were estimated separately for each species, and “·” indicates that the parameter is constant. Absence of γ(·) in the model notation implies that γ = 1 and absence of r(S) implies r(S) = p(S). “Lr” refers to the covariate scent lure persistence; and “Cam” refer to camera trap models. (DOCX) [file pone.0164271.s003.docx]

Table S1. The 16 models constructed and examined for each species pair.

| Model |
| --- |
| ψ(S)*p*(S) |
| ψ(S)*p*(S)*r*(S) |
| ψ(S)γ(.)*p*(S) |
| ψ(S)γ(.)*p*(S)*r*(S) |
| ψ(S)*p*(S+Lr) |
| ψ(S)*p*(S+Lr)*r*(S+Lr) |
| ψ(S)γ(.)*p*(S+Lr) |
| ψ(S)γ(.)*p*(S+Lr)*r*(S+Lr) |
| ψ(S)*p*(S+Cam) |
| ψ(S)*p*(S+Cam)*r*(S+Cam) |
| ψ(S)γ(.)*p*(S+Cam) |
| ψ(S)γ(.)*p*(S+Cam)*r*(S+Cam) |
| ψ(S)*p*(S+Lr+Cam) |
| ψ(S)*p*(S+Lr+Cam)*r*(S+Lr+Cam) |
| ψ(S)γ(.)*p*(S+Lr+Cam) |
| ψ(S)γ(.)*p*(S+L*r*+Cam)*r*(S+Lr+Cam) |
