# Supplementary material for: Spatial Co-Occurrence and Activity Patterns of Mesocarnivores in the Temperate Forests of Southwest China
Source: PLoS One. 2016 Oct 10;11(10):e0164271. doi: 10.1371/journal.pone.0164271 (PMC5056745; doi:10.1371/journal.pone.0164271)
Supplement: S2 Table — (DOCX) [file pone.0164271.s004.docx]

Table S2. Number of survey locations, survey efforts, and detections (number of locations which detected the species) for each species in the remote camera survey from 2004 to 2015 in Minshan Mountains, Southwest China.

|  | Wanglang | Huangtuliang | Laohegou | Total |
| --- | --- | --- | --- | --- |
| No. Locations | 207 | 170 | 118 | 495 |
| Camera-days | 8,958 | 5,707 | 8,648 | 23,313 |
| Species |  |  |  |  |
| Masked palm civet *Paguma larvata* | 34 (15) | 54 (45) | 48 (43) | 136 (121) |
| Leopard cat *Prionailurus bengalensis* | 53 (48) | 44 (42) | 24 (22) | 121 (112) |
| Yellow-throated marten *Martes flavigula* | 54 (53) | 21 (21) | 26 (24) | 101 (98) |
| Siberian weasel *Mustela sibirica* | 75 (72) | 49 (43) | 116 (96) | 240 (211) |
| Hog badger *Arctonyx collaris* | 110 (87) | 43 (39) | 53 (42) | 206 (168) |
| Asiatic golden cat *Catopuma temminckii* |  |  | 33 (12) | 33 (12) |
| Chinese ferret badger *Melogale moschata* |  |  | 1 (1) | 1 (1) |
| Red fox *Vulpes vulpes* | 2 (2) |  |  | 2 (2) |
