# Supplementary material for: Spatial Co-Occurrence and Activity Patterns of Mesocarnivores in the Temperate Forests of Southwest China
Source: PLoS One. 2016 Oct 10;11(10):e0164271. doi: 10.1371/journal.pone.0164271 (PMC5056745; doi:10.1371/journal.pone.0164271)
Supplement: S3 Table — (DOCX) [file pone.0164271.s005.docx]

Table S3. Number of detections, camera days in each month for all five species and *χ*^2^-test results on detection distribution to camera-days in the remote camera survey from 2004 to 2015 in Minshan Mountains, Southwest China.

| Species | Month | | | | | | | | | | | | *χ*^2^-test |
| --- | --- | --- | --- | --- | --- | --- | --- | --- | --- | --- | --- | --- | --- |
|  | 1 | 2 | 3 | 4 | 5 | 6 | 7 | 8 | 9 | 10 | 11 | 12 |  |
| Masked palm civet | 0 | 0 | 3 | 24 | 11 | 16 | 22 | 24 | 18 | 10 | 8 | 0 | $\text{}_{11}^{\text{2}}\text{ = 44.33}$, *P* < 0.001 |
| Leopard cat | 3 | 2 | 1 | 7 | 9 | 17 | 24 | 19 | 17 | 12 | 9 | 2 | $\text{}_{11}^{\text{2}}\text{ = 24.68}$, *P* = 0.010 |
| Hog badger | 0 | 3 | 7 | 20 | 33 | 40 | 31 | 25 | 35 | 9 | 3 | 2 | $\text{}_{11}^{\text{2}}\text{ = 54.29}$, *P* < 0.001 |
| Siberian weasel | 14 | 14 | 19 | 14 | 15 | 16 | 37 | 42 | 28 | 26 | 11 | 15 | $\text{}_{11}^{\text{2}}\text{ = 41.60}$, *P* < 0.001 |
| Yellow-throated marten | 14 | 4 | 4 | 8 | 15 | 8 | 12 | 4 | 7 | 9 | 8 | 8 | $\text{}_{\text{11}}^{\text{2}}\text{ = 31.62, }\text{P}\text{ < 0.001}$ |
| Camera-days | 1108 | 902 | 1256 | 2064 | 2848 | 2785 | 2627 | 2405 | 2613 | 2282 | 1610 | 813 |  |
